# Epigenetic modifications by polyphenolic compounds alter gene expression in the hippocampus

**DOI:** 10.1242/bio.035196

**Published:** 2018-07-03

**Authors:** Tal Frolinger, Francis Herman, Ali Sharma, Steven Sims, Jun Wang, Giulio Maria Pasinetti

**Affiliations:** 1 Department of Neurology, Icahn School of Medicine at Mount Sinai, New York, NY 10029, USA; 2 Geriatric Research, Education and Clinical Center, James J. Peters Veterans Affairs Medical Center, Bronx, NY 10468, USA

**Keywords:** Epigenetic modification, Polyphenols, Synaptic plasticity, DMR, Methylation

## Abstract

In this study, we developed an experimental protocol leveraging enhanced reduced representation bisulphite sequencing to investigate methylation and gene expression patterns in the hippocampus in response to polyphenolic compounds. We report that the administration of a standardized bioavailable polyphenolic preparation (BDPP) differentially influences methylated cytosine patterns in introns, UTR and exons in hippocampal genes. We subsequently established that dietary BDPP-mediated changes in methylation influenced the transcriptional pattern of select genes that are involved in synaptic plasticity. In addition, we showed dietary BDPP mediated changes in the transcriptional pattern of genes associated with epigenetic modifications, including members of the DNA methyl transferase family (*DNMTs*) and the Ten-eleven translocation methylcytosine dioxygenases family (*TETs*). We then identified the specific brain bioavailable polyphenols effective in regulating the transcription of *DNMTs*, *TETs* and a subset of differentially methylated synaptic plasticity-associated genes. The study implicates the regulation of gene expression in the hippocampus by epigenetic mechanisms as a novel therapeutic target for dietary polyphenols.

## Abstract (precis)

**Summary:** The health benefits of dietary polyphenols may be due to their ability to change epigenetic marks in the brain. More studies will clarify how polyphenols from diet can improve mental health.

## INTRODUCTION

Epigenetic modifications of the genome are a critical mechanism that controls the expression and types of genes transcribed from DNA. Within the brain, epigenetic modifications orchestrate the development (Schneider et al., 2016) and plasticity of synapses (Bongmba et al., 2011). Polymorphisms of genes that facilitate specific epigenetic modifications are associated with the formation of improper synapses and increase ones susceptibility to develop psychiatric disorders (Murphy et al., 2013). Differentially methylated regions (DMRs) of DNA are defined by the presence or absence of 5-methylcytosine (5mc) groups within the DNA template. The methylation status of cytosine residues in DNA are dependent upon the activity of epigenetic modifiers, such as by DNA methyl transferases (*DNMTs*) or Ten-eleven translocation methylcytosine dioxygenases (*TETs*). These epigenetic modifications are known to regulate gene expression in a region specific manner. Methylation of cytosine residues found in gene promoter regions is associated with suppression of gene expression (Schübeler, 2015). However, evidence to date has yet to establish a consistent relationship between the methylation of intronic, exonic, or untranslated regions (UTR) and the expression pattern of the gene’s corresponding proteins.

Previous studies have established that dietary polyphenols alter the epigenetic characteristics of DNA by regulating the enzymatic activity of *DNMTs* (Paluszczak et al., 2010) and histone deacetylases (Chung et al., 2010). For example, recent evidence suggests that bioavailable metabolites derived from dietary BDPP, such as malvidin glucoside (Mal-Gluc), decrease the expression of the inflammatory cytokine IL-6 from peripheral blood mononuclear cells, in part through mechanisms involving inhibition of cytosine methylation in intronic regions of the of IL-6 intron gene (Wang et al., 2018). Here we report standardized bioavailable polyphenolic preparation (BDPP) differentially influenced methylation patterns in introns’, UTR and exons' cytosine residues in hippocampal genes associated with brain plasticity and their concurrent transcriptional patterns of gene expression. In addition, we found BDPP-mediated regulation of the transcription of epigenetic modifiers, including *TETs* and *DNMTs* in the hippocampus.

The BDPP is composed of a complex composition of polyphenol compounds, which yield a variety of bioavailable derivatives following metabolism *in vivo* (Vingtdeux et al., 2010; Wang et al., 2015, 2014). Based on this, in combination with our preliminary BDPP pharmacokinetic studies (Ho et al., 2013), we further demonstrate individual polyphenol metabolites regulate epigenetic modifiers, ultimately influencing the expression of hippocampal genes associated to synaptic plasticity. Our results implicate epigenetic modifications altering gene expression as a novel therapeutic approach for treatment with dietary polyphenols.

## RESULTS

### BDPP-treatment influences the expression of methylation-related epigenetic modifying genes

**Fig. 1.**
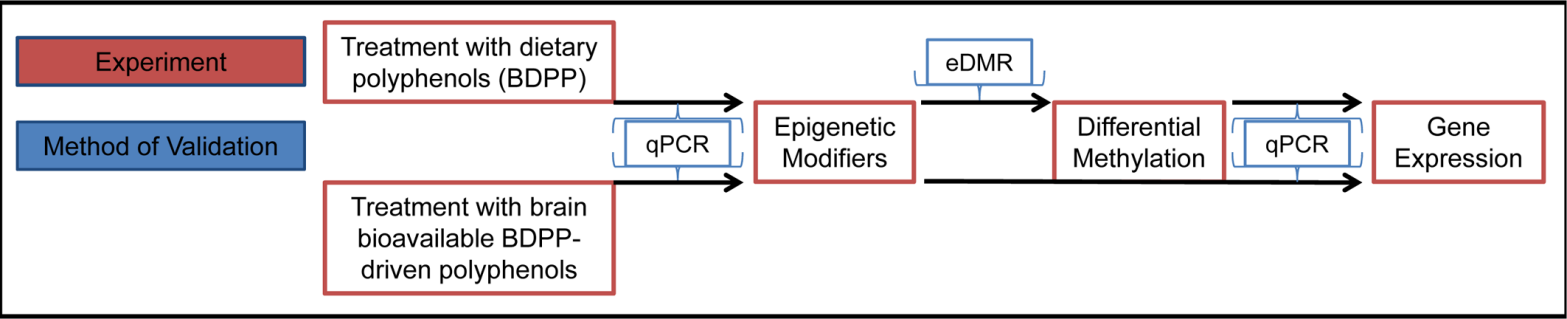
**BDPP treatment alters the expression of epigenetic modifying genes in the hippocampus of *C57BL/6* mice.** Fold change of mRNA expression of *DNMT1*, *DNMT3A*, *DNMT3B*. *TET1*, *TET2* and *TET3* in hippocampal extracts from BDPP treated mice (BDPP) relative to each in those from vehicle treated control mice (ctrl), assessed by qPCR. Expression was normalized to that of the housekeeping gene *HPRT*. Data are means±s.e.m. of 5­–11 mice in each condition (*P<0.05, **P<0.005 unpaired two-tailed *t*-test).

**Fig. 2.**
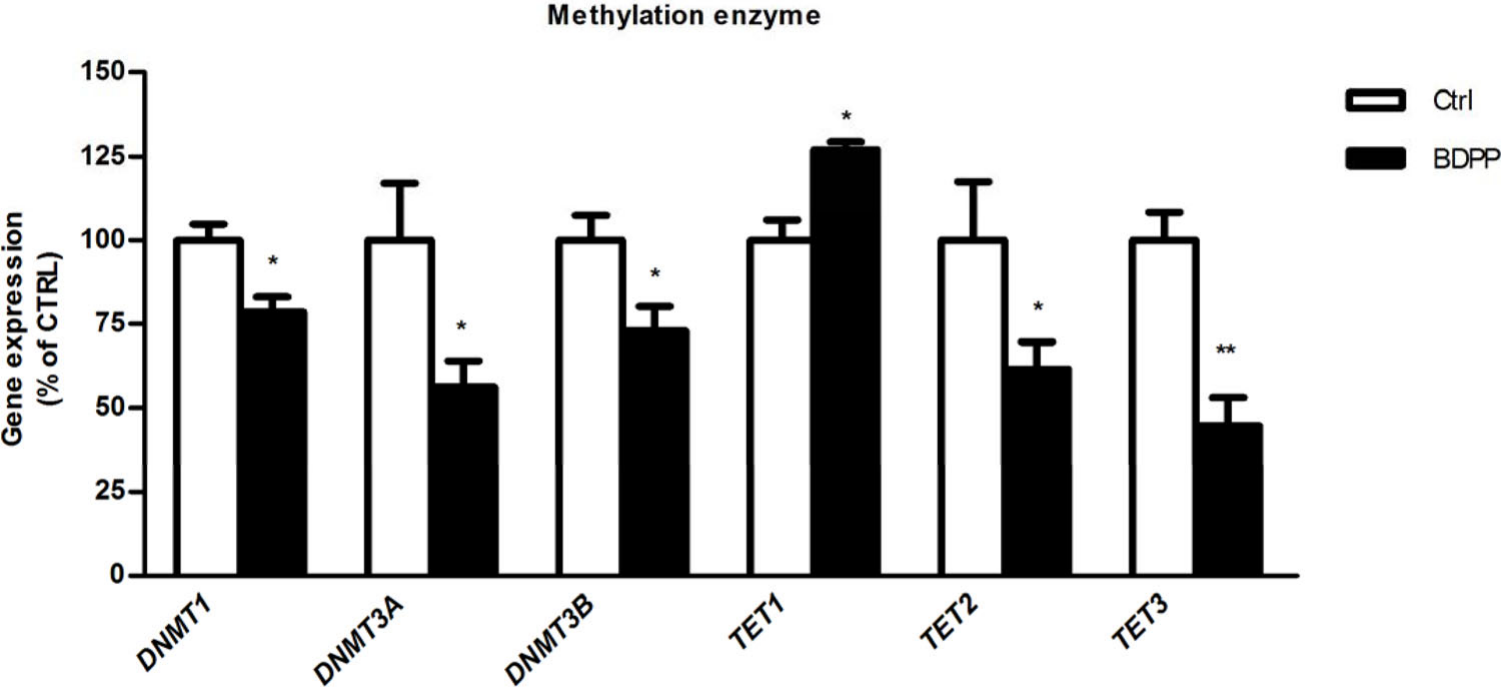
**BDPP treatment alters the expression of genes with differentially regulated DMRs in the hippocampus of *C57BL/6* mice.** (A) Fold change of mRNA expression of genes with DNA hypomethylated DMRs following BDPP administration; *ABPP2*, *ATG7*, *OCM*, *FIGF*, *ENOPH1*, *EIF4G*, *CCRL2*, *CHI3L1*. (B) Fold change of mRNA expression of genes with DNA hypermethylated DMRs following BDPP administration; *GRB10*, *RGS9*, *ITPKA*, *NDUFB9*, *CAMK2A*, *BRD4*. Expression was normalized to that of the housekeeping gene *HPRT*. Data are means±s.e.m. of 6–12 mice in each condition (*P<0.05, **P<0.005, unpaired two-tailed *t*-test).

In order to test whether dietary BDPP can contribute to synaptic plasticity through epigenetic mechanisms, *C57BL6* mice were randomly grouped into two groups: vehicle treated (control, ctrl) and BDPP treated (BDPP). Following two weeks' treatment, the hippocampus was isolated for DNA total RNA extraction (Fig. 1). In a first set of studies using real-time PCR, we quantified the expression of the epigenetics modifiers *DNMTs* and *TETs*, enzymes that are important for adding or removing methyl-groups to or from the DNA, respectively (Rasmussen and Helin, 2016; Robert et al., 2003; Robertson et al., 1999). We found BDPP treatment significantly reduced the mRNA expression of *DNMT1*, *DNMT3A DNMT3B*, *TET2*, and *TET3* and significantly increased the mRNA expression of *TET1* in the hippocampus as compared to ctrl (BDPP versus ctrl, *P*<0.05, Fig. 2). These results suggest BDPP-mediated activation of the DNA methylation machinery.

### Differential methylation of genes in the hippocampus of mice treated with BDPP

Based on the observation that dietary BDPP influences the methylation status of genes, we initiated a genome-wide methylation profile analysis using the RRBS technology followed by differential methylation analysis. Comparing BDPP to ctrl, we found 15 genes with differentially methylated DNA sequences. The DMRs ranged in length between ∼30 nucleotides to ∼300 nucleotides and were found on many different chromosomes. Among these DMRs, the relative amount of methylated CpG was found to be significantly reduced in six genes, while in nine genes the amount was found to be significantly increased in the BDPP treatment group as compared to ctrl (Table 1).

**Table 1.**
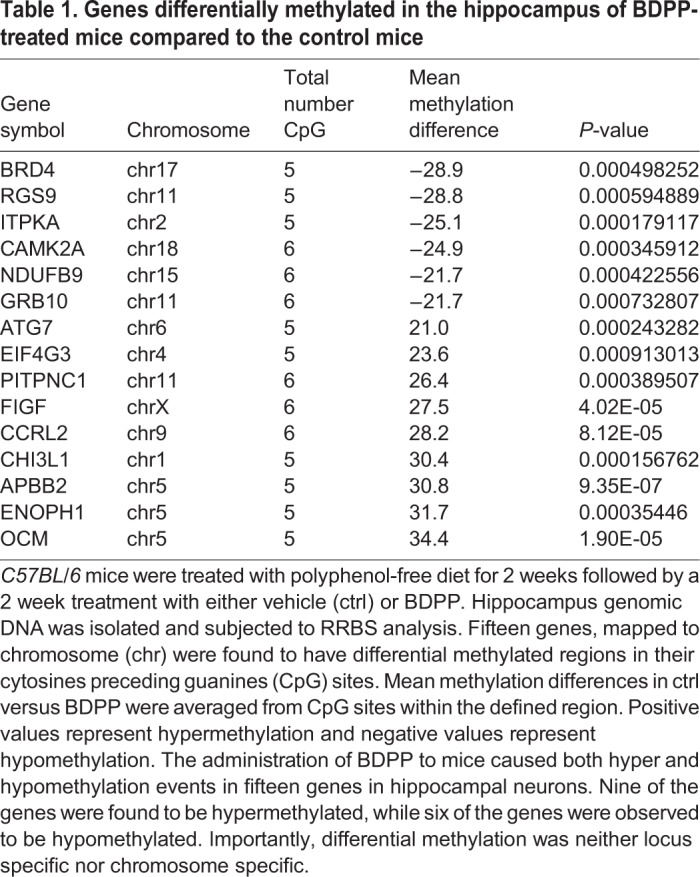
**Genes differentially methylated in the hippocampus of BDPP-treated mice compared to the control mice**

### Gene expression of differentially methylated genes in the hippocampus by BDPP

**Fig. 3.**
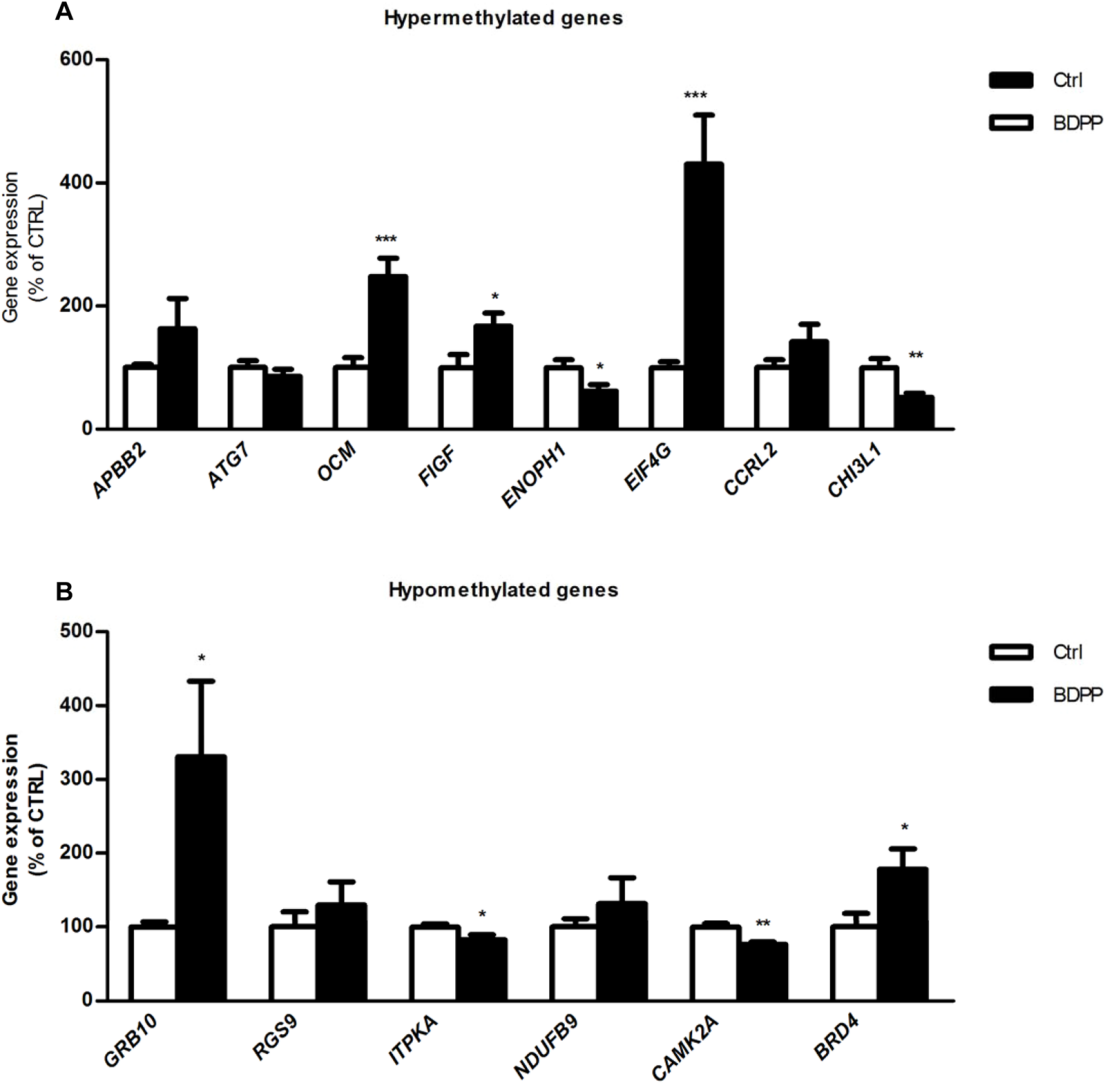
**Specific phenolic metabolites alter the expression of epigenetic modifying genes.** Primary neurons cell cultures were treated with malvidin-glucoside (MAL) or quercetin-3′-O-glucuronide(Q-GLUC) or delphinidin-3-*O*-glucoside (DEL) cyanidin-3-*O*-glucoside (CYA) or resveratrol (RES) or resveratrol-3′-*O*-glucuronide (R-GLUC) at concentration of 100 nM, or with the phenolic acids 3-(3′-hydroxyphenyl) propionic acid (HPP) or 3-hydroxybenzoic acid (HBA) at concentration of 2 μM for 24 h and compared to DMSO treated cells. Polyphenols and doses were chosen according to previously conducted brain bioavailability studies (Table 1). Cells were washed once with cold PBS and subjected to RNA isolation. Fold change of mRNA expression of epigenetic modifying genes: (A) *DNMT1*, (B) *DNMT3B*, (C) *TET1*, (D) *TET2* and of genes with differentially regulated DMRs, (E) *GRB10*, (F) *ITPKA*, (G) *CAMK2A* and (H) *APBB2* were assessed by qPCR. Expression was normalized to that of housekeeping gene *HPRT*. Data are means±s.e.m. of 4–5 samples in each condition (*P<0.05, **P<0.005, ***P<0.0005 unpaired two-tailed *t*-test).

Since transcription can be a function of CpG DNA methylation, we next quantified the gene expression of genes containing DMRs in the hippocampus of mice from BDPP and ctrl groups by qPCR. Among the genes with DMRs that were significantly hypermethylated in BDPP when compared to ctrl, we found a significantly increased mRNA expression of *OCM*, *FIGF* and *ElF4G* and significantly reduced the mRNA expression of *ENOPH1* and *CHI3L1* in the BDPP group, as compared to ctrl (Fig. 3A, BDPP versus ctrl, *P*<0.05). Among the genes with DMRs that were significantly hypomethylated in in BDPP when compared to ctrl, we found a significant increase in the expression of Grb10 and Brd4 and a significant decrease in the expressions of *ITPKA* and *CAMK2* in the BDPP group, as compared to ctrl (Fig. 3B, BDPP versus ctrl, *P*<0.05). Although the majority of the DMRs were found in the intronic region, DMRs were also found in coding regions and one was found in the untranslated region (UTR).The DMRs location, differential methylation in the DMRs and the expression of these specific genes are summarized in Table 2.

**Table 2.**
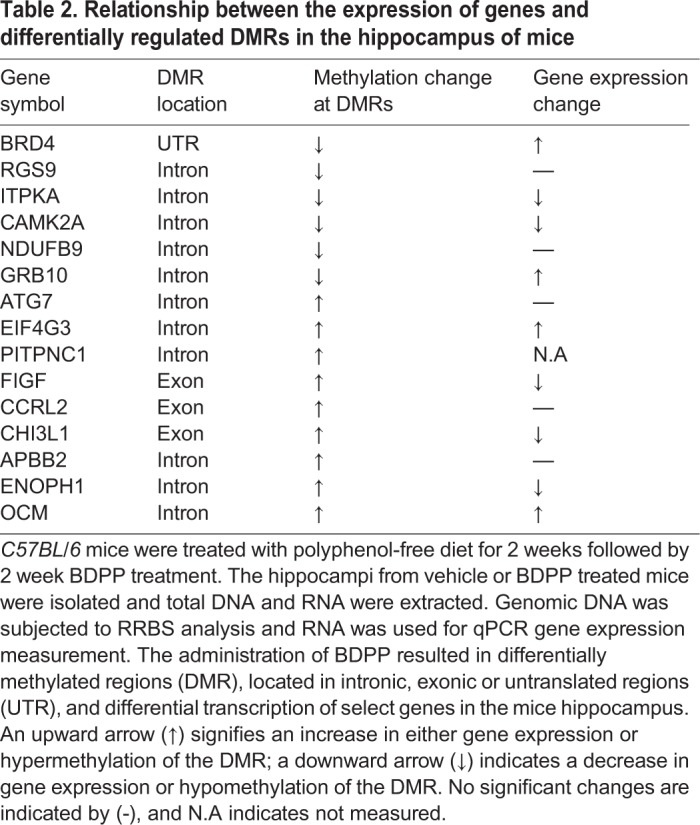
**Relationship between the expression of genes and differentially regulated DMRs in the hippocampus of mice**

### Specific polyphenol metabolites alter the expression of epigenetic modifying genes and differentially methylated genes

High-throughput bioavailability studies indicated that select BDPP derived polyphenolic metabolites accumulate in the brain following dietary BDPP treatment (Wang et al., 2015, 2014) (Table 3). To screen for metabolites that alter the expression of epigenetic modifying genes and differentially methylated genes, we treated primary embryonic mouse cortico-hippocampal neuron cultures with brain bioavailable polyphenol metabolites and measured mRNA expression of the epigenetic modifiers *DNMT1, DNMT3B, TET1, TET2* and selected differentially methylated genes *GRB10, ITPKA, CAMK2A,* and *ABPP2*. The select differentially methylated genes were chosen based on their contribution to synaptic plasticity (Guénette et al., 2017; Kim and Whalen, 2009; Shonesy et al., 2014; Xie et al., 2014).

**Table 3.**
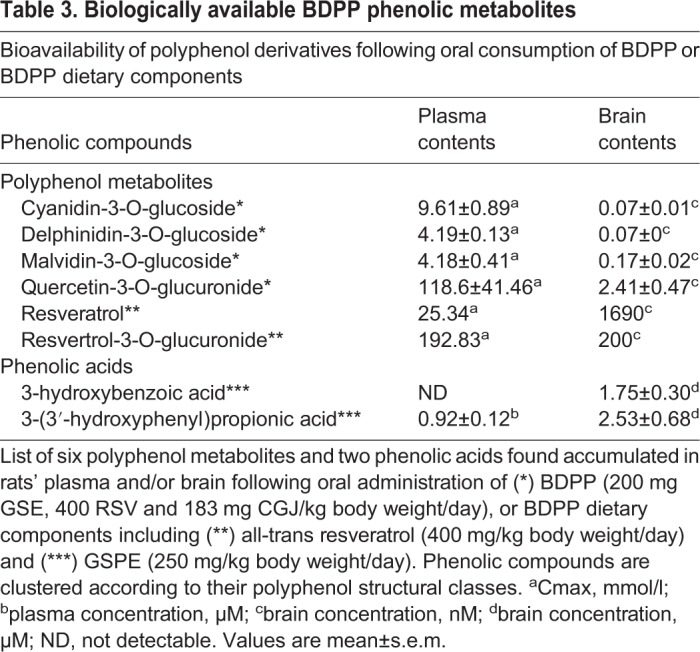
**Biologically available BDPP phenolic metabolites**

**Fig. 4.**
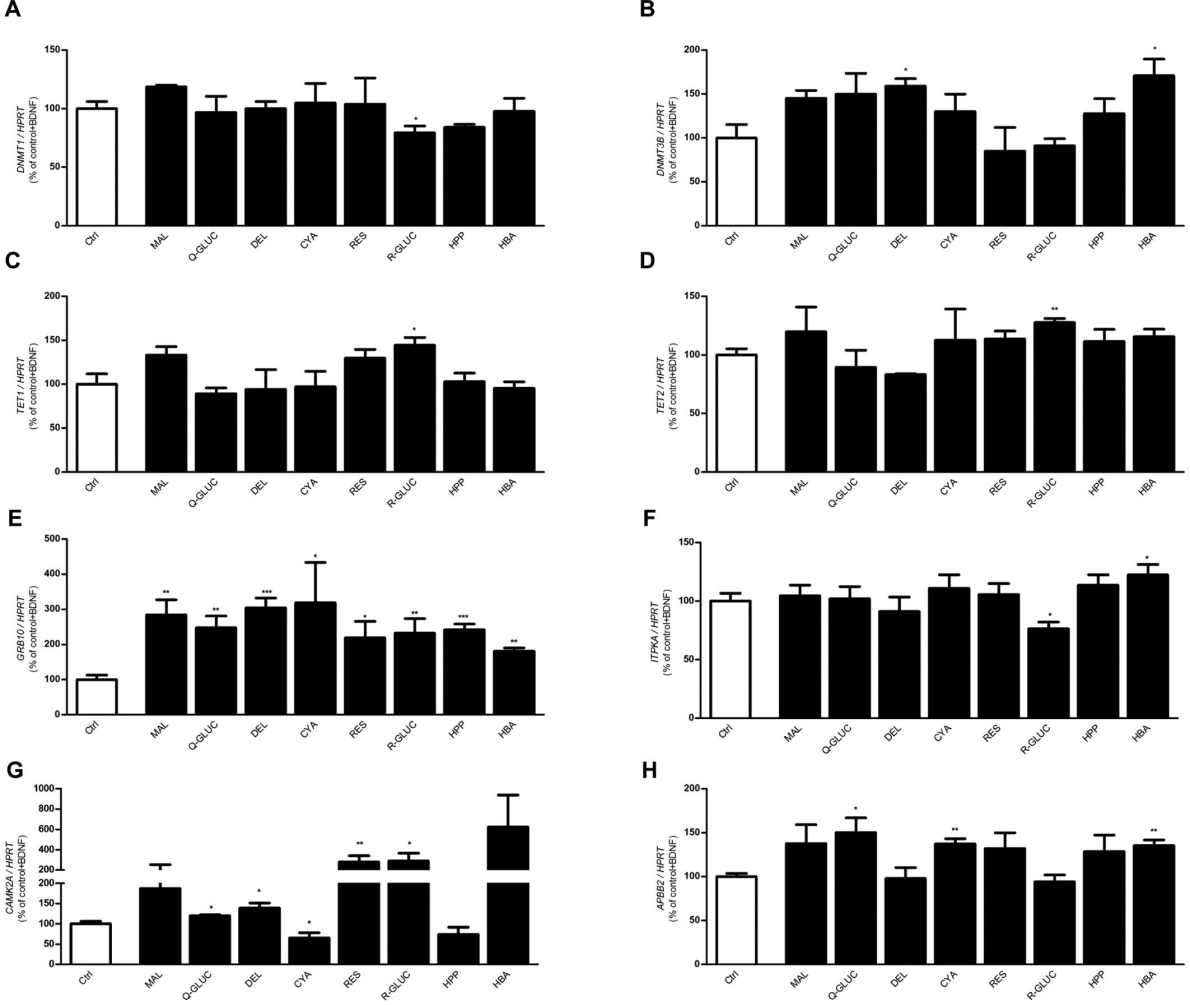
**Pleiotropic effects of BDPP-driven polyphenol metabolites.** Brain bioavailable BDPP-driven polyphenol metabolites (M) may have pleiotropic activity on different neural pathways. The net effect of the metabolites on neural pathways may provide positive (P1) or negative (P2) reinforcements, or their effects may cancel to yield no change in phenotype (P3). (*P<0.05, **P<0.005, ***P<0.0005 unpaired two-tailed t-test)

We found that compared to DMSO treated ctrl, primary embryonic mouse cortico-hippocampal neuron treated with R-GLUC had decreased expression of *DNMT1* (Fig. 4A, R-GLUC versus ctrl, *P*<0.05) and increased expression of *TET1* (Fig. 4C, R-GLUC versus ctrl, *P*<0.05) and TET2 (Fig. 4D, R-GLUC versus ctrl, *P*<0.05). In addition, treatment with DEL and HBA increased expression of *DNMT3B* (Fig. 4B, DEL, HBA versus ctrl, *P*<0.05). These results suggest BDPP-driven brain bioavailable polyphenols contribute to the activation DNA methylation machinery. We then examined the expression of differentially methylated genes that associate with synaptic plasticity. The effect of the selected brain-bioavailable phenolic compounds on gene expression is summarized in Table 4. We found all brain-bioavailable phenolic metabolites significantly increase the expression of *GRB10* in primary embryonic mouse cortico-hippocampal neurons (Fig. 4E, phenolic metabolites versus ctrl, *P*<0.05) compared to DMSO treated ctrl. Treatment with brain-bioavailable polyphenol metabolites (e.g. MAL, Q-GLUC, DEL, CYA, RES, R-GLUCC), but not phenolic acids (e.g. HBA, HPP), significantly increase the expression of *CAMK2A* (Fig. 4G, MAL, Q-GLUC, DEL, CYA, RES, R-GLUCC versus ctrl, *P*<0.05). In addition, treatment with R-GLUC decreased the expression of *ITKPA* (Fig. 4F, R-GLUC versus ctrl, *P*<0.05) and treatment with Q-Gluc or CYA or HAB increased the expression of *ABPP2* (Fig. 4H, Q-GLUC, CYA, HBA versus ctrl, *P*<0.05).

**Table 4.**
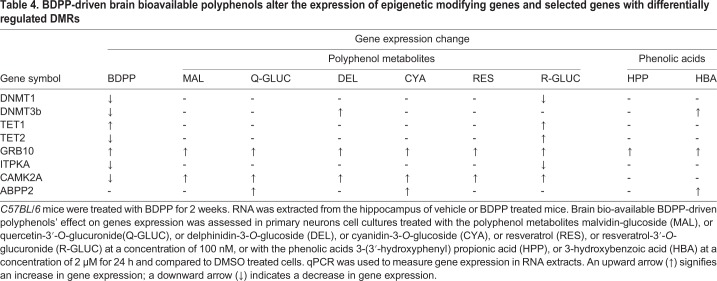
**BDPP-driven brain bioavailable polyphenols alter the expression of epigenetic modifying genes and selected genes with differentially regulated DMRs**

The inconsistent manners in which individual polyphenol metabolites alter gene expression suggest an additive or cancelation effect of different metabolites combinations.

## DISCUSSION

Epigenetic regulation of gene expression plays a critical role in orchestrating neurobiological pathways. The disruption of epigenetic networks is implicated as the source for a number of human brain disorders including autism, major depressive disorder and schizophrenia (Egger et al., 2004; Small et al., 2011). Hippocampal function in particular is susceptible to alterations in epigenetic mechanisms, which results in deficiencies in long term memory (Levenson and Sweatt, 2005; Sigurdsson and Duvarci, 2016) and synaptic plasticity (Yu et al., 2015). We have previously reported that dietary BDPP is effective in protecting against impaired performance in hippocampus-dependent cognitive tasks while the subject is experiencing conditions such as sleep deprivation, stress, and neurodegeneration (Pasinetti, 2012; Wang et al., 2012, 2014; Zhao et al., 2015). The principal objective of our study was to therefore explore the impact of BDPP on DNA methylation and the resultant gene expression in the hippocampus. We established that supplementation with dietary BDPP caused the differential expression of epigenetic modifiers, which are involved in the addition or removal of methyl groups from DNA cytosine residues. Through epigenetic profiling of hippocampal DNA, we present a list of hippocampal genes that had differential methylation of CpG sites following administration of BDPP and show that a number of these genes exhibit a concurrent change in their mRNA expression pattern. Furthermore, we identified specific brain bioavailable polyphenol metabolites that caused differential expression of both epigenetic modifiers, as well as a subset of the differentially methylated genes.

The methylation architecture of DNA is initially established by *de novo* DNA methyltransferases *DNMT3A* and *DNMT3B* (Okano et al., 1999), and then maintained during DNA replication and in senescence cells by the maintenance methyltransferase *DNMT1* (Robert et al., 2003). In order to maintain the steady state equilibrium of methylated/non-methylated CpGs, active DNA demethylation is initiated by *TET1* (Guo et al., 2011), TET2 (Ko et al., 2010) and TET3 (Li et al., 2014). Our finding that BDPP decreased the expression of *DNMT3A*, *DNMT3B*, *DNMT1*, which was concurrent with an increase in the expression of *TET1* and a decrease of *TET2* and *TET3* in the hippocampus, indicate BDPP may elicit genome-wide changes in methylation patterns through altering the ratio of *DNMTs* to *TETs*. Alterations to the ratio of epigenetic modifiers skew the steady state of methylated DNA CpG sites to hypermethylated or hypomethylated states (Pastor et al., 2013). In support of this principal, we show that BDPP treatment resulted in the hypermethylation of nine genes and the hypomethylation of six genes in the hippocampus. The differential methylation of genes induced by BDPP was non-specific in regards to the location in the gene; differential methylation was observed in intronic, exonic, as well as UTR regions. Only nine of the differentially methylated genes had simultaneous changes to their mRNA expression pattern. Separate mechanisms may therefore be involved in regulating gene transcription, such as the affinity of transcription factors for regulatory binding domains (Zaret and Carroll, 2011), cis-regulatory elements (Wittkopp and Kalay, 2011), or histone acetylation (Lawrence et al., 2016). Furthermore, hypermethylation or hypomethylation of CpG sites in a gene did not predict gene expression. Previous studies suggest that gene expression may be a function of the location of methylation within a gene. While increased methylation of gene promoter regions decreases gene expression (Schübeler, 2015), there is no defined or consistent relationship between methylation of intronic (Unoki and Nakamura, 2003), exonic (Jones, 1999) or UTR regions (Eckhardt et al., 2006; Reynard et al., 2011) and gene expression. For example, while methylation of upstream exon regions proximal to the 5′ transcription start site decreased gene expression (Brenet et al., 2011), the methylation of downstream exonic regions paradoxically increases gene expression (Jones, 1999; Kuroda et al., 2009). Our studies similarly found that hypermethylation of exonic regions resulted in either decreased gene expression or no corresponding change. In addition, hypermethylation and hypomethylation of intronic CpG sites yielded decreases, increases or no change in gene expression.

The tenuous relationship between methylation of gene body regions and gene expression, as illustrated in our study, may reflect the putative role of CpG site methylation in determining splice variant production. Methylation of exonic regions and intronic regions can promote alternative splicing through regulating RNA polymerase inclusion of exons (Maunakea et al., 2013). The use of pan primers in our experiment may have masked the effects of methylation in mediating the production of specific splice variants. Methylation of gene body regions may also play a role in promoting chromatin structure (Choi, 2010). However, establishing a relationship between methylation and splice variants is beyond the scope of this study.

DNA methylation is crucial for memory formation, as demonstrated in a number of organisms (e.g. honey bees, mollusks and rodents) and learning paradigms (Zovkic and Sweatt, 2013). Tet-mediated DNA demethylation is involved in the regulation of long-term memory formation as well (Kaas et al., 2013; Rudenko et al., 2013). Our finding of BDPP-mediated alternation of *DNMTs* and *TETs* gene expression suggest a mechanism for BDPP beneficial effect on memory (Zhao et al., 2015). In addition, a subset of the hippocampal genes that were both differentially expressed and methylated, including *BRD4*, *CAMK2A*, *ENOPH1*, *GRB10*, *ITKPA* and *ABPP2* have been previously implicated as regulators of neuronal activity or synaptic plasticity (Guénette et al., 2017; Kim and Whalen, 2009; Shonesy et al., 2014; Xie et al., 2014). The differential expression of both epigenetics mediators and plasticity-related gene expression following supplementation with BDPP may therefore influence synaptic plasticity and implicates epigenetic mechanisms as a potential mediator of hippocampal function.

**Fig. 5.**
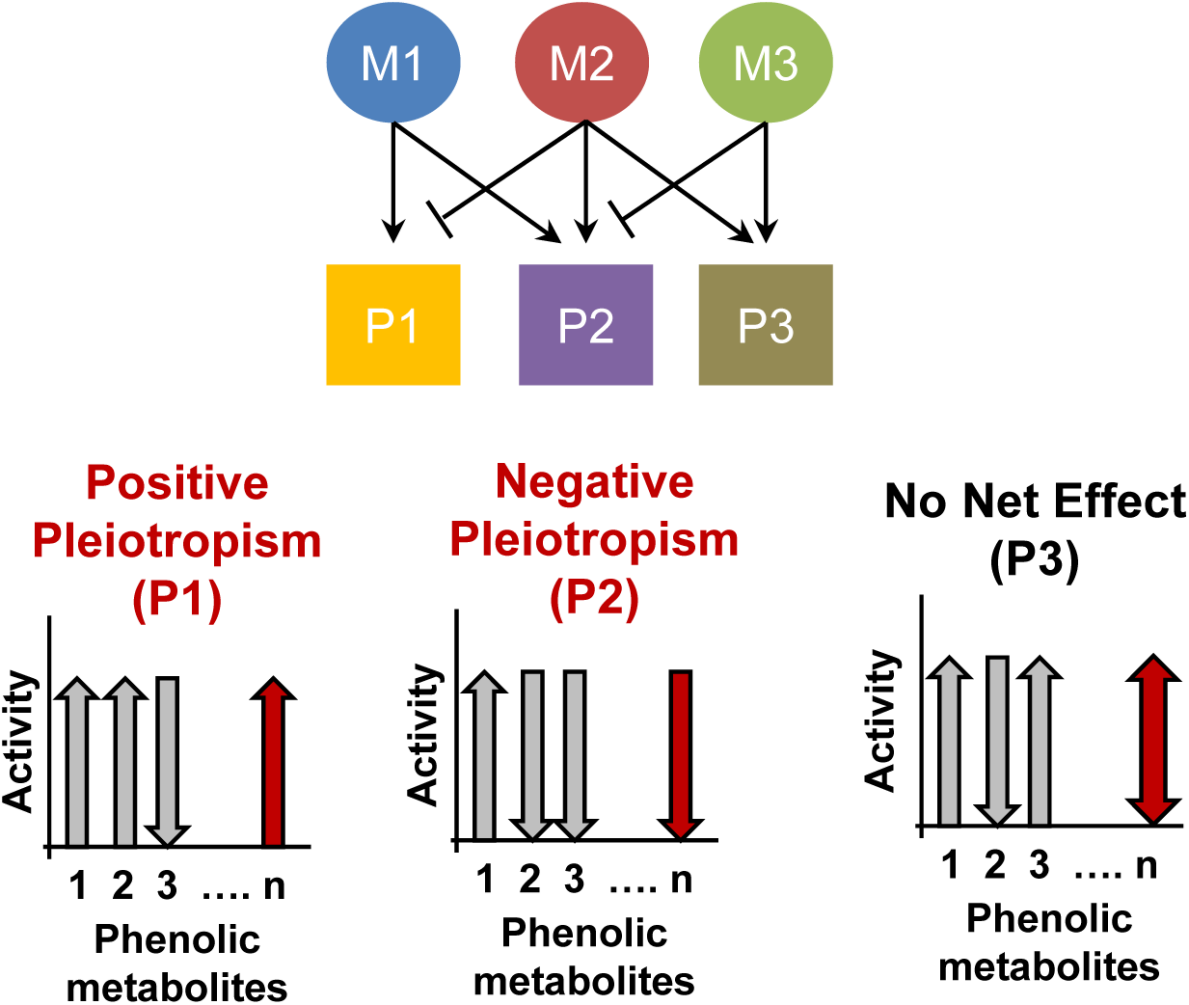
**Schematic of design of the experiments aimed to examine BDPP-mediated altered gene expression through epigenetic mechanisms.** *C57BL/6* mice were treated with polyphenol-free diet for 2 weeks followed by a 2 week BDPP treatment. The hippocampus was isolated and total DNA and RNA were extracted. Primary embryonic cortico-hippocampal neuronal cultures were treated with specific brain bioavailable BDPP-driven polyphenol metabolites prior to RNA extraction. Mice genomic DNA was subjected to RRBS analysis. Mice and primary embryonic cortico-hippocampal neuronal cultures RNA was used for qPCR gene expression measurements of epigenetic modifying genes and genes with differentially regulated DMRs.

We showed the brain-bioavailable polyphenolic metabolite R-GLUC can alternate the expression of the epigenetic modifiers *DNMT1*, *TET1* and *TET2* in primary neuronal cultures suggesting its ability to alter DNA methylation. Previous studies have showed the polyphenol metabolite MAL inhibition of DNA methylation effect through increasing histone acetylation (Wang et al., 2018), suggesting the specific brain bioavailable polyphenols may modulate DNA methylation through mechanisms different than *DNMTs* and *TETs*. In support with other studies showing the ability of specific polyphenol compounds to mediate the expression of genes involved in synaptic plasticity (Hsieh et al., 2012; Zhong et al., 2012) we showed that, when separately administered, the polyphenolic metabolites R-GLUC or MAL have either an increased effect, or no effect on the gene expression of the genes associated with synaptic plasticity, such as *GRB10*, *ITPKA*, *CAMK2A*, and *ABPP2*. Our results suggest that the net effect of BDPP on epigenetic mechanisms of gene expression is a result of the pleiotropic nature of the BDPP-derived bioavailable polyphenol metabolites and their cumulative effect on gene expression, which may be to promote, decrease or cause no change (Fig. 5). However, pleotropic effects of the combinations of polyphenol metabolites should be further investigated to better understand their interactions' contribution to genes' expression of both epigenetic modifiers and synaptic plasticity related genes.

Collectively, our results demonstrate that the administration of a dietary polyphenol preparation to mice alters the methylation status of the CpG islands of 15 genes in the hippocampal formation. Changes in gene methylation in the hippocampus occurred simultaneously with the differential expression of epigenetic modifiers in the *TET* and *DNMT* classes. An epigenetic mechanism may therefore be responsible for the observed changes in the mRNA expression of genes in the hippocampus that are associated with synaptic plasticity. Future studies will continue to investigate BDPP mediated differential gene expression via epigenetic modification as a mechanism for resilience against hippocampal-dependent cognitive dysfunction. Given the safety and tolerability of BDPP, our preclinical study has provided a basis for the potential translational application of dietary polyphenol compounds in promoting resilience to cognitive deficits by targeting epigenetic mechanisms.

## MATERIALS AND METHODS

### Materials

Polyphenol-free diet (AIN-93G) was purchased from Research Diets, Inc. (New Brunswick, USA). Food-grade resveratrol was purchased from ChromaDex (Irvine, USA). GSPE was purchased from Supplement Warehouse (UPC 603573579173, Bolingbrook, USA). One lot of the resveratrol and one lot of the GSPE were used for this particular study and were stored at 4°C in the dark. Concord purple grape juice (Welch Foods Inc., Concord, USA), Malvidin-3-O-glucoside, cyanidin-3-O-glucoside, delphinidin-3-O-glucoside, quercetin-3′-O-glucuronide, resveratrol-3′-O-glucuronide (Extrasynthesis, Genay Cedex, France), 3-hydroxybenzoic acid, 3-(3′-hydroxyphenyl) propionic acid, (Sigma-Aldrich) were obtained commercially. All tested compounds were analyzed by LC-MS and archived as previously reported (Vingtdeux et al., 2010; Wang et al., 2012) in compliance with NCCIH Product Integrity guidelines.

### Animals

*C57BL6/J* male mice (*Mus musculus*), *n*=24, were purchased from Jackson's laboratory at 12 weeks of age and group housed (five mice per cage) in the centralized animal care facility of the Center for Comparative Medicine and Surgery at the Icahn School of Medicine at Mount Sinai. All animals were maintained on a 12:12 h light/dark cycle with lights on at 07:00 h, in a temperature-controlled (20±2°C) room. All mice were allowed to adapt to the new environment for at least 2 weeks and were tested at 4–5 months old. For assessing BDPP effects mice were randomly assigned to vehicle-treated control group (*n*=12 per group) or BDPP-treated groups (*n*=12 per group). The calculated daily intake of GSE was 200 mg/kg body weight (BW), resveratrol was 400 mg/kg BW and the total polyphenols from juice extract was 183 mg/kg BW^6^. Mice were given BDPP delivered through their drinking water for 2 weeks prior to the experiment and the drinking solution was changed once every 2 days. Mice were euthanized with CO_2_ and hippocampi from each hemisphere were separately dissected, gently rinsed in ice-cold PBS and snap-frozen and stored at −80°C until further analyses. For all experiments, mice body weight and food consumption were assessed once a week (data summarized in Fig. S1). Liquid consumption was assessed every 2 days. Mice maintenance and use were approved by the Mount Sinai Animal Care and Use Committee.

### DNA and RNA extraction

For molecular investigation of BDPP effect, mice were euthanized with CO_2_ following 2 weeks of treatment. Hippocampi from each hemisphere were separately dissected, gently rinsed in ice-cold PBS and snap-frozen on dry ice for DNA and RNA studies. DNA and RNA from mouse hippocampus were simultaneously extracted from homogenized tissue using the Qiagen AllPrep DNA/RNA kit according to the manufacturer's instructions. Samples were stored at −80°C before further use. Total RNA from primary embryonic cortico-hippocampal neuronal cultures was isolated and purified using RNeasy Mini Kit (Qiagen) according to the manufacturer's instructions. Total RNA was eluted with nuclease-free water. The optical density (OD) ratio of 260/280 was measured using Nanodrop spectrophotometer (PeqLab Biotechnology, Erlangen, Germany) and ranged between 1.9 and 2.1. RNA samples were stored at −80°C before further use.

### Gene expression

In this study 1 µg of total hippocampal RNA and 400 ng of cells' RNA were reverse transcribed with a SuperScript first-strand III kit (Invitrogen). Real-time PCR were performed to confirm or identify genes of interest. Gene expression was measured in four replicates by quantitative RT-PCR using Maxima SYBR Green master mix (Fermentas, Waltham, USA) in ABI Prism 7900HT. Hypoxanthine phosphoribosyltransferase (*HPRT*) expression level was used as an internal control. Data were normalized using the 2^−ΔΔCt^ method (Livak and Schmittgen, 2001). Levels of target gene mRNAs were expressed relative to those found in ctrl mice hippocampal tissue for *in vivo* studies and to untreated cells+BNDF induction for the cell cultures studies and plotted in GraphPad Prism. The primers used for the gene expression studies are listed in Table 5.

**Table 5.**
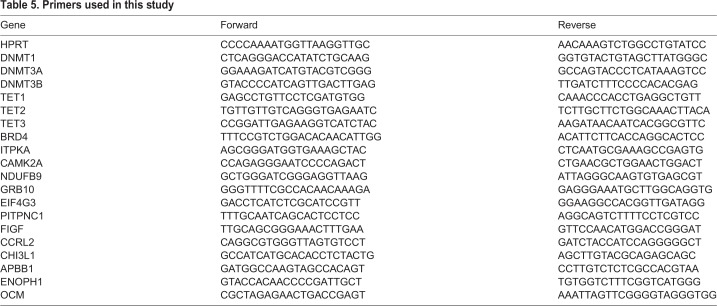
**Primers used in this study**

### Enhanced reduced representation bisulphite sequencing (eRRBS)

RRBS libraries, sequencing, data alignment and methylation calls were generated at the Epigenomics Core, Weill Cornell Medicine. Briefly, 50 ng of genomic DNA were digested with 100 U of MspI (New England Biolabs, Ipswich, USA) and end-repaired/A-tailed using Kapa Hyper Prep kit (Kapa Biosystems, Wilmington, USA). After ligation of Illumina-sequencing compatible indexes, DNA was purified using a 1X Agencourt AMPure XP bead clean up (Beckman Coulter, Inc., La Brea, USA). Bisulfite conversion was carried out using the Zymo EZ DNA kit (Zymo Research, Irvine, USA) using the following program: 55 cycles: 95°C 30 s, 50°C 15 min, 4°C ∞. Libraries were amplified 17 cycles using Uracyl+ Ready mix (KK2801, Kapa Biosystems, Wilmington, USA). The resulting libraries were normalized to 2 nM and pooled according to the desired plexity, clustered at 6.5 pM on single read flow cell and sequenced for 50 cycles on an Illumina HiSeq 2500. Base call files generated from the sequencer were demultiplexed and converted to FASTQ files using the CASAVA (CASAVA, RRID: SCR_001802) software. These reads were then aligned to the mm10 build of the mouse genome and post-processed to produce methylation calls at a base pair resolution using a previously described pipeline developed at the Epigenomics Core, Weill Cornell Medicine.

### Differential methylation analysis

Cytosines preceding guanines (CpG) sites within the defined region in the resulting RRBS data were then interrogated for methylation patterns and differential methylation (q-value<0.01 and methylation percentage difference of at least 25%) using methylKit package in R software (methylKit, RRID:SCR_005177). The differential methylation data was then queried for differentially methylated regions (DMRs) using eDMR . Downstream statistical analyses and plots were generated using the R software environment for statistical computing.

### Mouse primary embryonic cortico-hippocampal neuronal cultures

Primary cortico-hippocampal neurons were prepared from E15 *C57BL6/J* mouse (*M. musculus*) embryos as previously described (Wang et al., 2007). Embryonic brain tissue was mechanically triturated and centrifuged. Neurons were seeded onto poly-D-lysine-coated 6-well plates and cultured in the serum-free chemically-defined neurobasal medium, supplemented with 2% B27, 0.5 mM L-glutamine and 1% penicillin-streptomycin (Invitrogen). The absence of astrocytes (<2%) was confirmed by the virtual absence of glial fibrillary acidic (GFAP) protein immunostaining (data not shown).

### Effect of select bioavailable polyphenols treatment on gene expression

Following 14 days being cultured, neurons in the vehicle control group were treated with DMSO (Sigma-Aldrich) and neurons in treatment groups were treated with Malvidin-glucoside (Mal), cyanidin-3-*O*-glucoside (CYA), delphinidin-3-*O*-glucoside (DEL), quercetin-3′-O-glucuronideand (Q-gluc), Resveratrol (RES), resveratrol-3′-*O*-glucuronide (Res-gluc) at the concentration of 100 nM or with the phenolic acids 3-hydroxybenzoic acid (HBA) and 3-(3′-hydroxyphenyl) propionic acid (HPP) at the concentration of 2 μM for 24 h. DMSO dilutions ranged from 10^5^ to 10^7^. Cells were stimulated with 15 ng/μl of BDNF (Sigma-Aldrich, Cat: B3795) for 1 h and then washed once with cold PBS and subjected to RNA isolation. mRNA expression of *TET1, TET2, DNMT1, DNMT3B, GRB10, ITPKA, APBB2* and *CAMK2A* was assessed by RT-PCR. Potential cytotoxic effects of the individual polyphenols and their combination were tested using the quantitative colorimetric assay of LDH (CytoTox 96, Promega, Madison, USA).

### Overall statistics

All values are expressed as mean and standard error of the mean (s.e.m.). Unpaired two-tailed Student's *t*-tests with Welch's correction were used. In all studies, outliers (2 s.d. from the mean) were excluded and the null hypothesis was rejected at the 0.05 level. All statistical analyses were performed using Prism Stat program (GraphPad Software, Inc.).

## Supplementary Material

Supplementary information
