# Supplementary material for: Epigenetic modifications by polyphenolic compounds alter gene expression in the hippocampus
Source: Biol Open. 2018 Jul 3;7(10):bio035196. doi: 10.1242/bio.035196 (PMC6215408; doi:10.1242/bio.035196)
Supplement: Supplementary information [file biolopen-7-035196-s1.pdf]

Biology Open (2018): doi:10.1242/bio.035196: Supplementary information

A

Table 1S		Ctrl (n=12)				BDPP (n=12)				P (2-way ANOVA)					
										Treatment effect		Time effect		Treatment x Time	
		Week 1	Week 2	Week 3	Week 4	Week 1	Week 2	Week 3	Week 4	P	F (DFn,DFd	P	F (DFn,DFd	P	F (DFn,DFd
Body weight (gr)	Mean	28.691	28.250	29.625	30.675	28.275	28.608	29.891	30.850	0.862	0.03<br>1,22	<0.0001	9.67<br>3,66	0.871	0.24<br>3,66
	SEM	0.611	0.5315	0.543	0.5929	0.630	0.522	0.585	0.666						
Food consumption (gr)	Mean	3.850	3.462	3.296	3.429	3.633	3.182	3.183	3.216	0.152	3.12<br>1,4	0.048	3.52<br>3,12	0.968	0.08<br>3,12
	SEM	0.107	0.185	0.082	0.191	0.278	0.183	0.064	0.156						
Liquid consumption (ml)	Mean	4.836	5.59	5.14	3.925	4.707	5.41	4.42	4.276	0.699	0.15<br>1,21	0.027	3.24<br>3.63	0.699	0.48<br>3,63
	SEM	0.314	0.424	0.732	0.469	0.299	0.342	0.736	0.422						

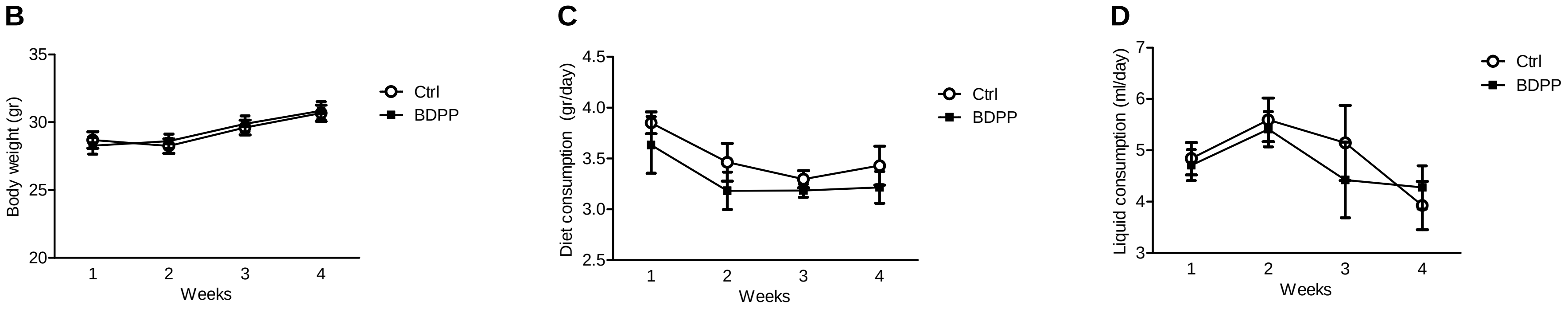

Fig. S1. Physiological monitoring data. A) Body weight (gr/week), diet consumption (gr/day) and liquid consumption (ml/day) of vehicle (Ctrl) or BDPP treated mice. Values are mean ± SEM. Repeated measures ANOVA was used to calculate *p* values. **B, C, D)** Graphic presentation of body weight gain (gr/week), diet consumption (gr/day) and liquid consumption (ml/day) across the 4-weeks treatment regimen. Data is mean ± SEM (Repeated measures ANOVA).

Biology Open • Supplementary information
